# Practical application of short-term intensive insulin therapy based on the concept of “treat to target” to reduce hypoglycaemia in routine clinical site

**DOI:** 10.1038/s41598-020-58574-7

**Published:** 2020-01-31

**Authors:** Koji Nakashima, Nobuhiro Okamura, Hayato Sanefuji, Hideaki Kaneto

**Affiliations:** 1 Department of Internal Medicine, Okamura Isshindow Hospital, 2-1-7 Saidaiji-Minami, Okayama, Okayama 704-8117 Japan; 2 Department of Diabetes, Endocrinology and Metabolism, Kawasaki Medical School, 577 Matsushima, Kurashiki, 701-0192 Japan; 3 Department of Cardiovascular Medicine, Okayama university Medical School, 2-5-1, Shikata, Kitaku, Okayama 700-8558 Japan; 4 Department of Pathology, Yamaguchi University Medical Graduate School, 1-1-1 Minami-Kogushi, Ube, Yamaguchi 755-0046 Japan

**Keywords:** Endocrinology, Medical research

## Abstract

The aim is to devise a new short-term intensive insulin therapy (N-SIIT) based on the concept of “treat to target” to avoid hypoglycaemia and was applied it to various diabetic state. We determined dosage of 1 basal and 3 bolus “treat” insulin based on “target” blood glucose level and changed each insulin dose by small units (2 units) every day for 2 weeks. We evaluated the effects of N-SIIT in 74 subjects with type 2 diabetes (male 45, female 29, 64.9 ± 16.6 years old, HbA1c 10.4 ± 2.6%). Glargine U300 (“treat”) and morning blood glucose level (“target”) was significantly correlated with increasing insulin dose and decreasing blood glucose level in day 1–7, indicating that insulin amount was determined by target blood glucose level and lowered next target blood glucose level. Remission rates were 67.3% (Hypoglycaemia rate 5.6 %) in N-SIIT and 47.3% (Hypoglycaemia rate 38.1%) in conventional SIIT. Required amount of insulin would be automatically determined, depending on each patient pathophysiology and life style. This method is pretty simple, flexible and cheap, and provides information about the dynamic pathophysiological alteration of insulin resistance and glucotoxicity from the profile of blood glucose levels and insulin shot.

## Introduction

Type 2 diabetes mellitus (T2DM) is a progressive disease which gradually reduces pancreatic beta-cell function such as insulin secretory capacity and increases insulin resistance in various insulin target tissues^1,2^. Recently, short-term intensive insulin therapy (SIIT) is recommended in the treatment of newly diagnosed T2DM to eliminate glucotoxicity, to reduce beta-cell overload (beta-cell rest effect), to support residual beta-cells and to enhance insulin sensitivity^3–18^.

In addition, pancreatic alpha-cell dysfunction may contribute to the metabolic dysfunction found in diabetic state, because post-prandial paradoxical hyperglucagonaemia leads to the elevation of blood glucose levels^19,20^. SIIT may also improve alpha-cell physiology^21–23^. Indeed, it is possible that stepwise addition of insulin leads to the reduction of hyperglucagonaemia.

The evidence of this treatment has been presented to prove benefits in the treatment of T2DM. However, in most SIIT studies, after SIIT they did not use any anti-diabetic agents and evaluated the duration of glycaemic remission^3–18^. As the results, glycaemic remission was temporary, especially when beta-cell function was markedly deteriorated after SIIT. Retnakaran *et al*. regarded SIIT as an induction therapy and sequential treatment with anti-diabetic agents as a maintenance therapy^24^. Several kinds of anti-diabetic agents such as metformin or GLP-1 receptor activator had been used for a maintenance therapy, but sometimes re-induction of insulin therapy was necessary^24–27^. We used conventional SIIT for the elimination of glucotoxicity in clinical practice and thought that SIIT was useful not only in newly diagnosed T2DM but also under many kinds of diabetes conditions to obtain good glycaemic control. However, hypoglycaemia was the most undesirable and harmful side effect of conventional SIIT^28–30^. It is well known that hypoglycaemia leads to various clinical problems such as acute coronary syndrome, fundus hemorrhaging and unaware hypoglycaemia. Therefore, in practical medicine, we should be very careful to avoid hypoglycaemia when treating diabetes.

In this study, to reduce the risk of hypoglycaemia, we devised the new SIIT (N-SIIT) simply based on the concept of “treat to target” by stepwise addition or reduction of small units of insulin (basically 2 units of insulin). Present “treat” insulin was determined by past “target” blood glucose level. We titrated independently four injections of one-basal and three-bolus insulin. We think that four glycaemic targets (5.0–7.2 mmol/L) prevent excess insulin dosage, leading to avoid hypoglycaemia. Also, it is likely that we can recognize the signals of glucotoxicity elimination and recover insulin sensitivity in each patient’s insulin–glucose profile, leading to discontinue insulin injection to avoid hypoglycaemia due to prolonged insulin therapy. In addition, in this study we propose the concept “free resistance day (FRD)” when we recovered insulin sensitivity and discontinued insulin injection.

We retrospectively analyzed blood glucose, HbA1c, C-peptide (CP), C-peptide index (CPI) after this new SIIT in subjects with T2DM. In this report, we show the data obtained in all subjects (74 cases) for remission induction therapy using SIIT.

In comparison of N-SIIT and conventional SIIT (C-SIIT), the data in 54 cases who received maintenance therapy in our out-patient clinic after N-SIIT were compared with those in 55 T2DM treated with C-SIIT and the maintenance therapy.

It is likely that required amount of insulin would be almost automatically and more easily determined with this method compared to conventional one. We think that this method has an advantage to reduce hypoglycaemia. We can get information about the relief of insulin resistance as well as glucotoxicity from the profile of blood glucose levels and insulin shot. We could use this dynamic pathophysiological alteration to obtain good glycaemic control in diabetic patients with lower risk of hypoglycaemia.

## Methods

### Subjects

Seventy four subjects with T2DM (male 45, female 29, age 64.7 ± 16.6 years old, HbA1c 10.4 ± 2.6%) (23 insulin users, 27 newly diagnosed T2DM subjects and 24 subjects using diabetic agents except for insulin (non-insulin diabetic agents (NIDA)) were admitted to our hospital since December 1, 2016 to September 30, 2019 and analyzed for dynamic alteration of blood glucose levels in this study. In order to evaluate HbA1c and C-peptide, we used 54 cases who were followed by maintenance therapy in our outpatient clinic. Fifty four subjects with T2DM (male 36, female 18, age 60.6 ± 16.8 years old, HbA1c 10.8 ± 2.4%) (16 insulin users, 19 newly diagnosed T2DM patients and 19 subjects using NIDA) were analyzed. In comparison of new SIIT (N-SIIT) and conventional SIIT (C-SIIT), we obtained the data in 55 T2DM patients who had been admitted to our hospital and received C-SIIT since March 1, 2001 to November 31, 2016 (male 28, female 25, age 72.4 ± 11.2 years old, HbA1c 9.7 ± 1.5%). We analyzed and compared the data between N-SIIT and C-SIIT group.

This research was approved by the research ethic board of Okamura Isshindow Hospital (No.18000151-2018 (1)) and was conducted in accordance with the Declaration of Helsinki. Signed inform consent was obtained from each patient.

### Measurement of blood glucose and serum C-peptide levels

Capillary blood glucose levels were measured by STAT STRIP Xpress (Nova Biomedical, Welthon, MA 02454, USA) on the tip of fingers. Serum C-peptide (CP) levels were measured by immunoezymometric assay (LumipulseG C-peptide, Fujirebio KK, 51 Hachioji, Tokyo 192-0031, Japan). C-peptide index (CPI) (ng/mg) was calculated by the formula: 100 × fasting CP (ng/ml)/fasting plasma glucose (mg/dL). We created 2 new parameters from the patient insulin-glucose profile to visualize dynamic glucose change in this new SIIT. First one was free insulin resistance day (FRD) to release insulin resistance (range 3–25 day) and second one was a sum of maximum basal-bolus insulin in a day (Max-Insulin) (range 20–162 units).

### Study protocol

On the first hospital day, medical history taking, physical examination and laboratory tests including fasting blood glucose and serum C-peptide, transaminase, lipids, HbA1c, urine tests and daily profile of blood glucose were carried out. Patients were consulted to an ophthalmologist to evaluate diabetic retinopathy. In hospital, patients take diabetes diet, which menu and calorie were individually provided for each patient by the nutritionist, and exercise using bicycle ergometer for 20 min after lunch under the instruction of exercise specialist.

Japanese Board Certified Diabetologists give the information of the patient to the diabetes treating team which is consist of registered nurses (Certified Diabetes Educator of Japan, CDEJ), registered dieticians (CDEJ), clinical pharmacists and exercise trainers and supervise the team and SIIT. Doctor in charge has to prescribe the first order of insulin dose and anti-diabetic agents after SIIT. When the specialist recognizes that each patient needs more than 2-unit elevation of insulin, the doctor changes it appropriately. The ward nurses perform daily measurement of blood glucose levels and registry on the table in Supplementary Fig. S1a, titration of insulin dose and insulin injection, and take care of the patients especially in view of dietary intake. It is obvious that when the patients cannot eat food enough, they have a higher risk of hypoglycaemia.

**Figure 1:**
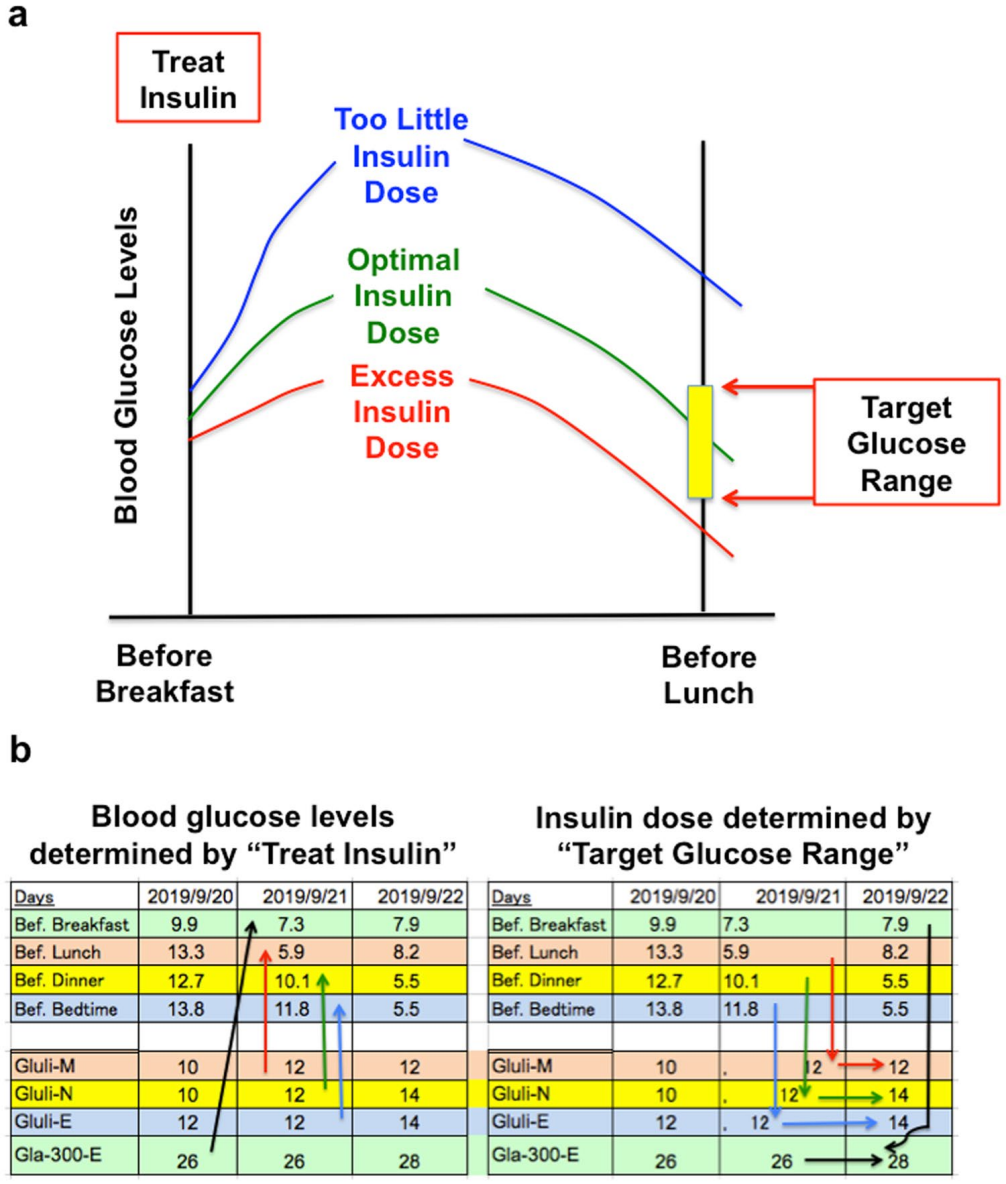
Relation between “treat” insulin and “target” glucose range. (**a**) Schematic illustration about blood glucose levels determined by insulin. (**b**) Blood glucose levels determined by “treat” insulin and insulin dose determined by “target” glucose range. Bef. Breakfast: Before breakfast, Bef. Lunch: Before Lunch, Bef: Dinner: Before Dinner, Bef. Bedtime: Before Bedtime. Gluli-M: morning glulisine, Gluli-N: noon glulisine, Gluli-E: evening glulisine, Gla-300-E: evening glarugine-300.

Forty % of initial total daily dose of 0.4–0.6 IU/kg insulin is delivered with Gla-300 before dinner as a basal insulin and 20% is applied with each glulisine before each meal as a bolus injection. In the following days, insulin dose is titrated according to the latest blood glucose level based on the concept of “treat to target” (Fig. 1a,b).

Relation of insulin and glucose level is as follows: Glu-M, which is injected before breakfast and works to reduce elevated blood glucose level by breakfast, determines glucose level before lunch (Fig. 1a,b). Similarly, Glu-N before lunch determines blood glucose level before dinner. Glu-E before dinner determines blood glucose level at the bedtime. Gla-300 injected before dinner determines fasting blood glucose level on the next day. Insulin which determines blood glucose level at pre-meal and bed time is defined as “treat” insulin, and blood glucose level which is determined by “treat” insulin is called “target” glucose. Real patient’s N-SIIT was presented in Supplementary Fig. S1. In C-SIIT, insulin dose was determined by sliding scale as shown in Supplementary Table. T1.

### Statistical analysis

Data with normal distribution were examined by Shapiro-Wilk test. Blood glucose and HbA1C levels were not normally distributed. Statistical analyses were done using non-parametric method. Wilcoxon’s signed rank tests and rank sum tests were applied for comparison of the data. In comparison of three groups, p values were adjusted by Bonferroni-Holm’s calculating formula. Associations of variables were assessed with Spearman’s rank correlation coefficient. We used JMP v 8 for statistic calculation.

## Results

**Figure 2:**
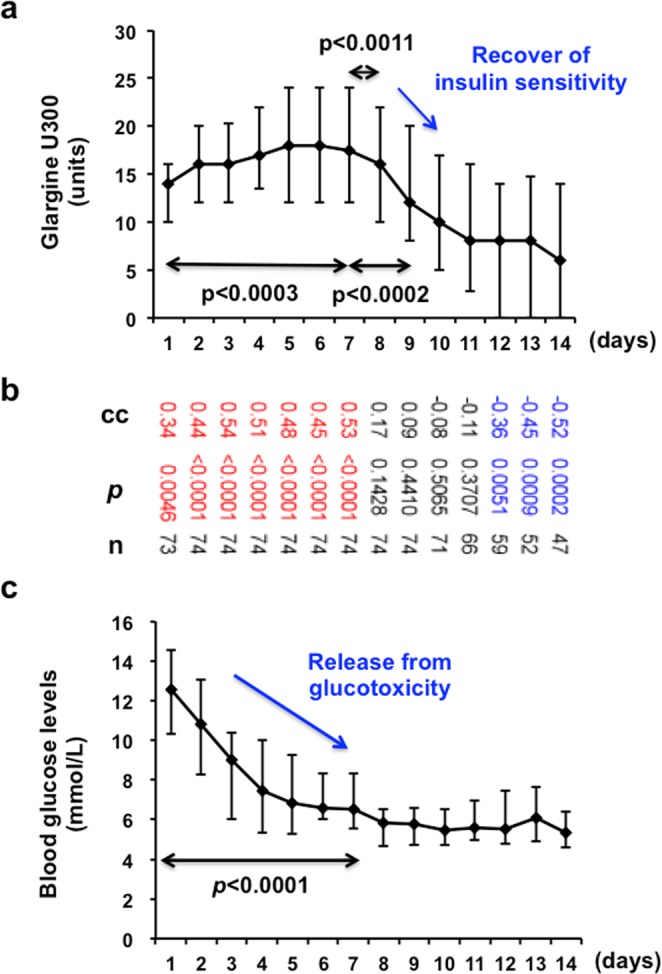
Profile of Injected Gla-300U dose (“treat” insulin) and morning blood glucose levels (“target” glucose) in N-SIIT. (**a**) Injected Gla-300 dose (median, Error bar: interquartile range (IQR)). An arrow indicates p value between day 1 and day 7, day 7 and day 8 and day7 and day 9 (Wilcoxon’s signed rank test n = 74). P values were adjusted by Bonferroni-Holm’s calculating formula for multiple comparison. (**b**) Correlation coefficient (CC) of Gla-300 and morning blood glucose levels in each day, and p value (P) (Spearman’s rank correlation coefficient). Numbers at the bottom: subject number. Red CC and P show significant co-relation. (**c**) Morning capillary blood glucose levels (median, IQR). An arrow indicates p value between day 1 and day 7 (Wilcoxon’s signed rank test, n = 74).

As shown in Fig. 2, there was close relation between evening Gla-300 dose (“treat”) and morning blood glucose level (“target”) in the daily glycaemic control by insulin. Median of Gla-300 dose elevated significantly till day 7, but after then it decreased. In addition, inter quarter range (IQR) became wider gradually (Fig. 2a). Blood glucose level decreased reciprocally to Gla-300 dose, but in day 8–14 blood glucose level did not decrease. IQR was smaller than those in day 1–6, and as the results, blood glucose level did not become lower than 3.9 mmol/L (Fig. 2c).

Decrease of glucose levels in day 7 reveals that glucotoxicity was canceled (Fig. 2c).

In day 8 and 9, we experienced that glucose level was acutely responded by change of “treat insulin” and recognized relief of insulin resistance and we had to reduce insulin dose (statistically lower insulin dose in day 8 and 9 than in day 7), indicating that the patient recovered insulin sensitivity. In performing this SIIT, we can get information about the relief of insulin resistance and glucotoxicity from the profile of glucose levels and insulin shot, and after then we can replace SIIT to maintenance therapy in FRD. This is a great benefit of this method for the prevention of hypoglycaemia due to long-term intensive insulin therapy.

Figure 2b shows correlation coefficient (CC) and p value between Gla-300 dose and morning blood glucose level. Number (n) of patients were decreased from 74 to 47 because 27 patients were discharged from this hospital after established remission from glucose toxicity by day 15. Gla-300 dose and morning glucose level (day 1–7) showed significant positive correlation. In day 8–11, there was no significant correlation. In day 13–14, there was negative correlation, probably because blood glucose level reached target range and Gla-300 dose decreased and finally insulin therapy was discontinued

**Figure 3:**
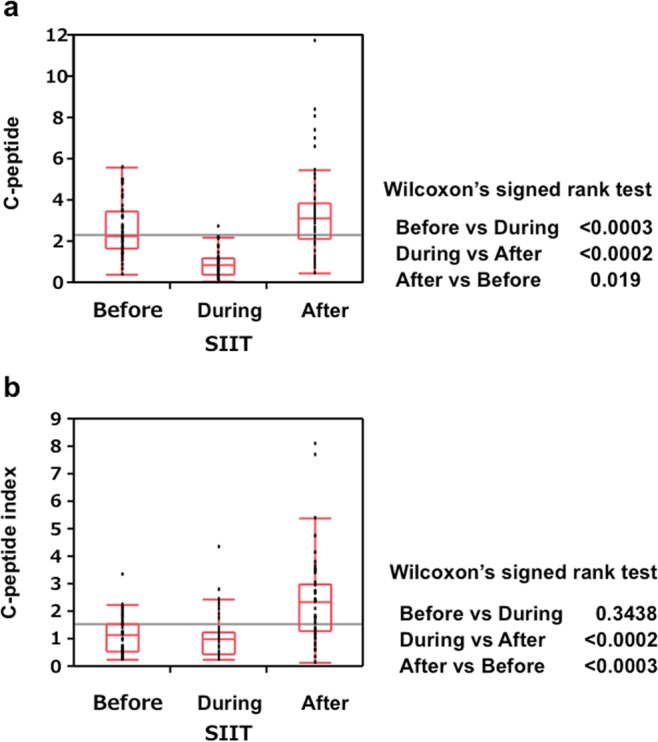
Box-and-whisker plots of C-peptide and C-peptide index before, during and after N-SIIT. Serum C-peptide (**a**) and serum C-peptide index (**b**) before SIIT, SIIT and after SIIT (Median, IQR). In comparison of three groups, P values were adjusted by Bonferroni’s calculating formula. Solid arrows show p values in Wilcoxon’s signed rank test. n = 54.

Figure 3 shows serum C-peptide (CP) and C-peptide index (CPI), before, during and after N-SIIT. CP decreased during SIIT but elevated after SIIT. CPI was lower before and during SIIT but elevated after SIIT. These data suggest that beta-cells might have a rest by injected insulin which decreased beta-cell insulin secretion by feedback mechanism (beta-cell rest effect).

**Figure 4:**
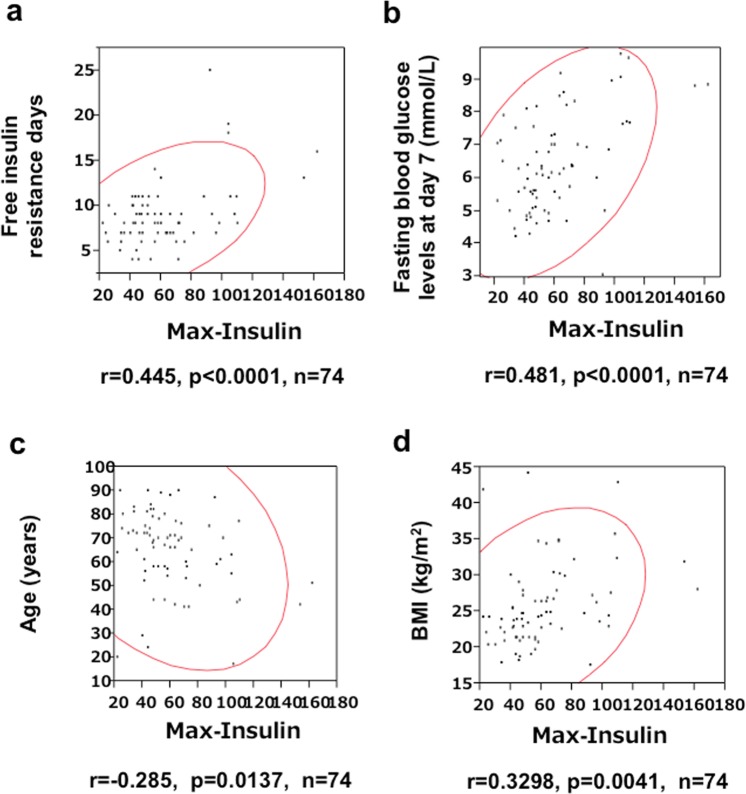
Scatcher plots of Max-Insulin and free insulin resistance day (FIRD) (**a**), fasting glucose levels at day 7 (**b**), age (**c**) and BMI (**d**). r, correlative coefficient; p, p value; n, number of subjects. Red curve: 0.95 probability ellipse.

Figure 4a showed significant correlation between FRD and Max-Insulin, suggesting that the patient who had stronger insulin resistance spent longer duration of SIIT and larger amount of insulin to achieve relief of insulin resistance. Max-insulin was correlated with morning glucose of day 7 (Fig. 4b), indicating that the patients with higher glucose needed large amounts of insulin. In Fig. 4c, inverse correlation between age and Max-inulin suggested that the younger patient needed larger amounts of insulin. Higher BMI needed larger insulin. These data in Fig. 4 sowed a dynamism in N-SIIT.

**Figure 5:**
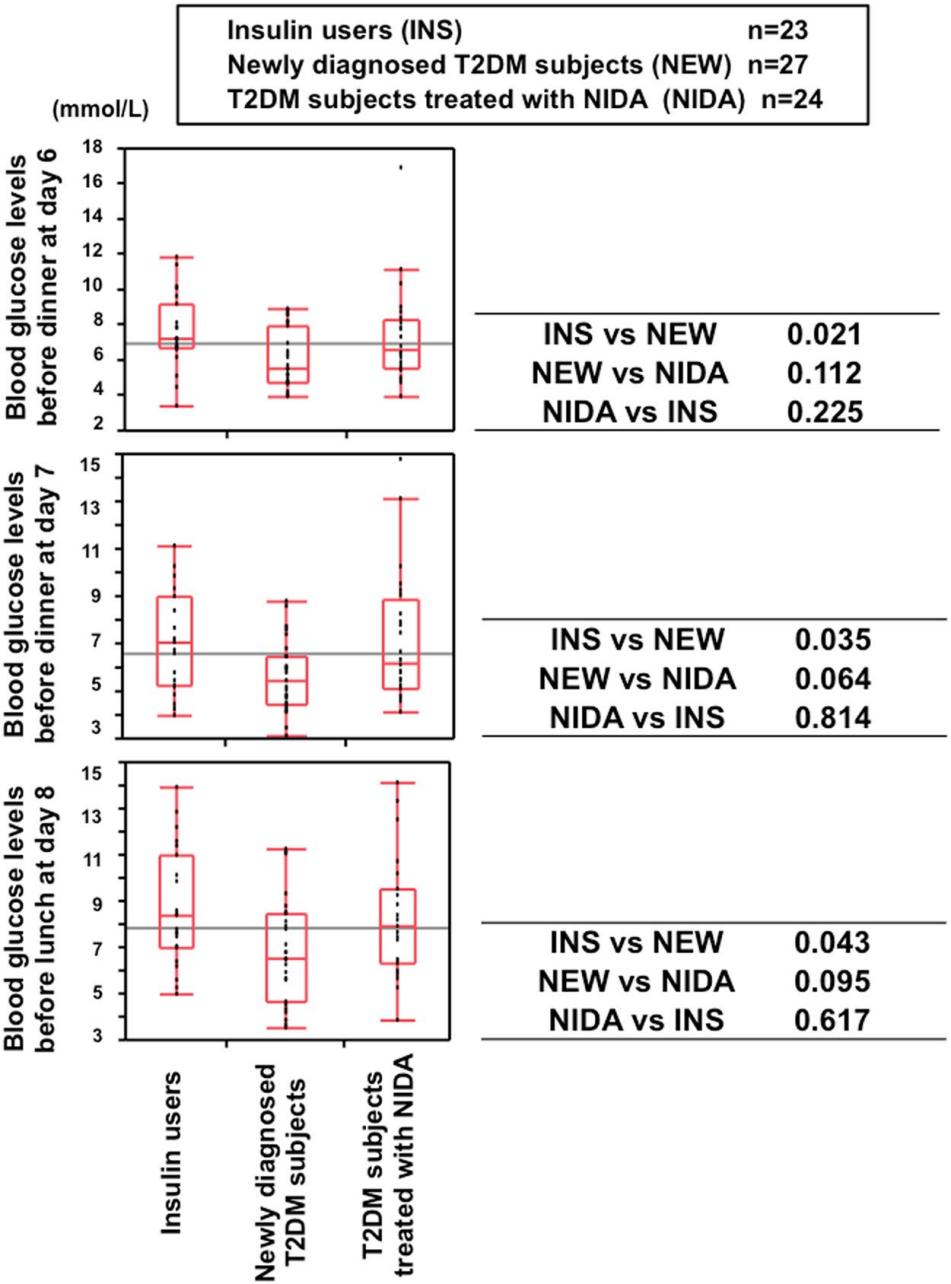
Box-and-whisker plots of blood glucose levels in insulin users (N = 23), newly diagnosed T2DM subjects (N = 27) and subjects using non-insulin diabetes agents (NIDA) (N = 24) before dinner at day 6 and day 7 and before lunch at day 8. Wilcoxon’s signed rank test was performed. p values were adjusted by Bonferroni-Holm’ equation.

In comparison of insulin users, newly diagnosed T2DM and subjects using NIDA (Fig. 5), blood glucose levels in newly diagnosed T2DM subjects were significantly lower than those in insulin users, although they were not different at the start of SIIT. In the end of N-SIIT it appeared that there was a sign of different ability of beta-cell activity among the groups.

**Figure 6:**
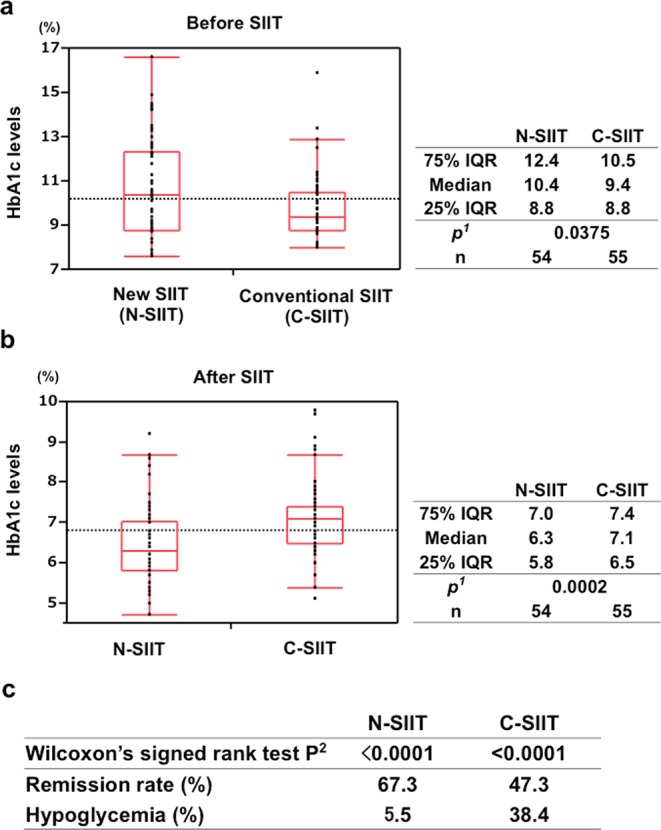
Box-and-whisker plots of HbA1c values in subjects treated with new SIIT (N-SIIT) and conventional SIIT (C-SIIT). (**a**) Before SIIT; (**b**) After SIIT. p^1^: p value of Wilcoxon’s rank sum test. p^2^: p value of Wilcoxon’s signed rank test.

HbA1c valued in subjects treated with N-SIIT and C-SIIT lowered respectively after SIIT (Fig. 6c). In comparison of N-SIIT and C-SIIT before and after SIIT, HbA1c values in N-SIIT group were higher than those in C-SIIT group before SIIT and was lower after SIIT (Fig. 6). Remission rate (67.3%) in N-SIIT group was higher than that (47.3%) in C-SIIT group. Furthermore, hypoglycaemic rate (5.6%) in N-SIIT group was lower than that (38.4%) in C-SIIT group. In N-SIIT, glucose less than 3.9 mmol/L were recorded in 3 patients’ glucose tables, day 7 dinner 3.5 mmol/L, day 6 bedtime 3.7 mmol/L and day 7 bedtime 3.7 mmol/L when the patients were near or in FRD without any hypoglycaemic symptoms.

**Figure 7:**
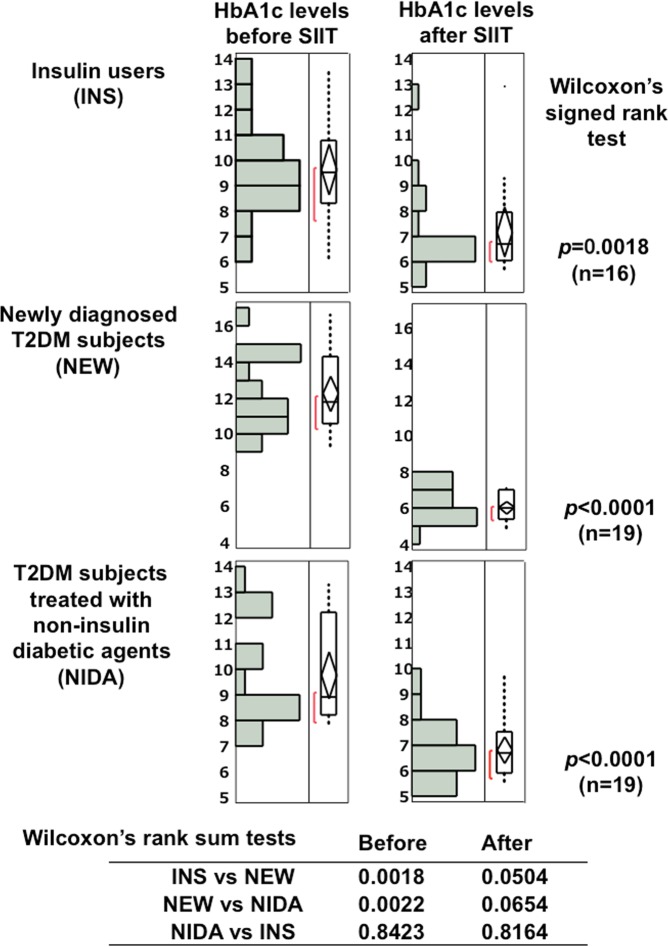
Histogram and box-and-whisker plots of HbA1c values before and after SIIT in insulin users (n = 16), newly diagnosed T2DM subjects (n = 19), subjects using non-insulin diabetic agents (NIDA) (n = 19).

In Fig. 7, HbA1c histograms and box-and-whisker plots of insulin users, newly diagnosed T2DM and subjects with NIDA before and after N-SIIT. Each group significantly improved in HbA1c after N-SIIT. Before SIIT, HbA1c values in newly diagnosed T2DM subjects was significantly higher than those in insulin users and subjects with NIDA. HbA1c values in these groups lowered without significant difference, indicating that HbA1c in three groups were titrated in similar HbA1c range. Insulin user and subjects using NIDA were treated in a similar way as newly diagnosed T2DM subjects.

We presented our real patients in Supplementary Data in order to show real patient treatments and the possibility of N-SIIT application in the routine clinical practice and described brief comments in Discussion.

## Discussion

T2DM is a progressive disease which gradually reduces pancreatic beta cell function such as insulin secretory capacity and increases insulin resistance in various insulin target tissues^1,2^. Decline in pancreatic islet beta cell function were explained by two components; (1) “reversible” metabolic components (e.g. glucotoxicity, lipo-toxicity and insulin resistance); (2). “irreversible” intrinsic components (e.g. loss of beta cell capacity/mass due to beta cell apoptosis). Each is independently contributing to the pathophysiological process of the disease.

Short-term (two to four week) intensive insulin therapy (SIIT) administered early in the course of T2DM acutely improves beta cell function by eliminating glucotoxicity, lipo-toxicity and insulin resistance^3–18^. Long-term intensive insulin therapy (LIIT) was used to compensate defective insulin to make physiological form of insulin secretion in the irreversible component of beta cell death. LIIT works as maintenance therapy in T2DM with irreversible component of beta cell death. Many treatment algorithms and clinical trials of T2DM with beta cell death have been proposed so far^31–34^. Basal-Bolus, Basal plus, Basal supporting oral therapy (BOT), premixed insulins and premixed insulin and GLP-1R agonist were reported to improve safety and efficacy of the patient treated mainly in out-patient clinic^35–42^.

On the other hands, SIIT is useful for newly diagnosed T2DM hospitalized for short term, as a remission induction therapy. The persistent decline of beta cell function in newly diagnosed T2DM patients cannot be prevented with routine hypoglycaemic strategies^2,17^. Basal-bolus insulin injection (SIIT) is applied as the first anti-diabetic agent in newly diagnosed T2DM. The evidence of this treatment has been presented to prove benefits in the treatment of T2DM^1,2^. We used basal-bolus SIIT for the elimination of glucotoxicity in clinical practice and devised new SIIT to reduce hypoglycemia and to improve efficiency based on the concept of “treat to target”.

“Treat” insulin amounts before each meal (3 glulisine and 1 glargine U300) were determined individually and independently according to previous blood glucose level which was determined by latest insulin (Fig. 1, Supplementary Fig. S1 and method section). This was repeated daily until when blood glucose levels reached within target glucose range (5.0–7.2 mmol/L). We thought that when glucose levels before each meal and bedtime were within 5.0–7.2 mmol/L, glucotoxicity was withdrawn and the amounts of insulin were adjusted to each patient’s own pathophysiology automatically. In other words, this is a new procedure to determine each amount of insulin, depending on each patient’s own pathophysiology and life style. After relief of glucotoxicity, “treat” insulin became more powerful and we had to reduce insulin dose to keep glycaemic target, indicating that the patients have recovery of insulin sensitivity. Also, in this study we proposed a new parameter “free insulin resistance day (FRD)”. This is important because we have to reduce insulin dose on FRD in each injection independently and discontinue N-SIIT, resulting to avoid hypoglycaemia due to prolonged insulin using.

In Fig. 3, C-peptides (CP) and C-peptide index (CPI) before, during and after SIIT were presented. CP during SIIT was significantly lower than those before and after SIIT and CP after SIIT was significantly higher than that before SIIT, suggesting that insulin secretion was accelerated before SIIT, suppressed during SIIT and recovered after SIIT. Beta-cells had a rest of insulin production and secretion by feedback action of SIIT. In Fig. 3b, CPIs before and during SIIT were not significantly different and CPI before SIIT was significantly lower than that of CPI after SIIT, suggesting that insulin secretion was not enough to decrease blood glucose levels before SIIT. We recognized this during SIIT, following FRD. Figure 4a showed significant correlation between FRD and Max-Insulin, suggesting that patients who had stronger insulin resistance spent longer duration of SIIT and larger amounts of insulin to achieve relief of insulin resistance. Max-insulin was correlated with fasting blood glucose levels at day 7 (Fig. 4b), indicating that patients with higher blood glucose levels needed larger amounts of insulin. In Fig. 4c, inverse correlation between age and Max-Insulin suggested that younger patients needed larger amounts of insulin and patients with higher BMI needed larger amounts of insulin.

In comparison of insulin users, newly diagnosed T2DM and subjects using NIDA (Fig. 5), blood glucose levels in newly diagnosed T2DM subjects were significantly lower than those in insulin users. It seemed that there was substantial difference in insulin secretory capacity in beta-cells among the groups. Taken together, this new SIIT provided the basic evidence of ability to contribute to the inadequately controlled diabetic patients. HbA1c values in subjects treated with new SIIT (N-SIIT) and conventional SIIT (C-SIIT) lowered respectively after SIIT. In comparison of N-SIIT and C-SIIT before and after SIIT, HbA1c of N-SIIT was higher than that of C-SIIT before SIIT and was lower after SIIT (Fig. 6). Remission rate of N-SIIT was higher than that of C-SIIT. Furthermore, hypoglycaemic rate of N-SIIT was lower than that of C-SIIT. These data clearly demonstrated that N-SIIT is much more superior compared to C-SIIT in a variety of points from the clinical point of view. In N-SIIT, glucose less than 3.9 mmol/L were recorded in 3 patients’ glucose tables without any hypoglycaemic symptoms, day 7 dinner; 3.5 mmol/L, day 6 bedtime; 3.7 mmol/L and day 7 bedtime; 3.7 mmol/L when the patients were near or in FRD, We confirmed the relief of insulin resistance.

In Fig. 7, HbA1c histograms and box-and-whisker plots of insulin users, newly diagnosed T2DM and subjects with NIDA before and after N-SIIT. Each group significantly improved in HbA1c after N-SIIT. Before SIIT, HbA1c values in newly diagnosed T2DM subjects was significantly higher than those in insulin users and subjects treated with NIDA. HbA1c in three groups were titrated in similar HbA1c range. These data indicate that SIIT is very useful to obtain good blood glucose control under a variety of conditions.

In conventional SIIT, present blood glucose level is used to determine insulin dose, but this method does not necessarily determine appropriate insulin dosage based on each subject pathophysiology. This point is completely different from our concept “treat to target”. Therefore, we believe that the relation of blood glucose levels and insulin dose in this method is much stronger than that in conventional sliding scale. As shown in Fig. 2, in the stage (day 1–7) of elevating insulin dose, blood glucose levels decreased reciprocally to the insulin dose elevation with significant correlation, but the correlation was lost in the stage of target range (day 8–12) and glucose level did not decrease, suggesting that this method has a great advantage to avoid hypoglycaemia, because glucose levels entered into the target range and “treat” insulin did not increase. Interestingly, correlation coefficient of day 13 and 14 was significantly inverse one. After blood glucose levels entered in the target range, “treat” insulin was reduced or replaced by NIDA, without aggravation of glycaemic control. Indeed, 27 patients discharged from the hospital by day 15, because they completed SIIT and started taking maintenance therapy.

In addition, it was reported that Gla-300 has lower possibility of hypoglycaemia than Gla-100 and insulin degrudec^43–46^. The peak of Gla-300 was reported to be lower than Gla-100 and presented 12–13 h later^46^. When we inject Gla-300 around 18:00, its peak concentration appears around 7:00 next day. We can monitor blood glucose levels when GLa-300 is the highest concentration in blood. Target glucose range is 5.0–7.2 mmol/L and this range is a little bit higher but is enough to cancel glucotoxicity.

It is known that pancreatic alpha-cell dysfunction brings out post-prandial paradoxical hyperglucagonaemia which elevates blood glucose levels and thereby contributes to the metabolic dysfunction found in diabetic state^19–23^. We recently reported that diabetic rats expressed GLP-1 receptor in alpha-cells and that GLP-1 analogue stimulated glucagon secretion from alpha-cells but such glucagon secretion was inhibited by simultaneously secreted insulin from beta-cells in paracrine fashion^47^. Therefore, it is possible that stepwise elevation of insulin dose in this SIIT suppresses glucagon secretion which leads to smooth lowering of blood glucose levels to the target range.

We would like to preset our real patients in Supplementary Data in order to show real patient treatments and the possibility of N-SIIT application in the routine clinical practice as follows.

As shown in Supplementary Fig. S1, Case 1 was hospitalized in our institution with acute cellulitis and inadequately controlled T2DM. We successfully treated her with antibiotics and N-SIIT. In general, infection has difficult problems in diabetic patients because both diseases worsen each other as a vicious circle. In this case, however, both infection and diabetes were appropriately cured by antibiotics and N-SIIT. We used antibiotics and SIIT in other patients with infection such as septicemia, pneumonia and urinary tract infection. Our SIIT was useful in such other diabetic patients together with infection. This method would be useful for many diabetic patients with a variety of situations such as patients who receive surgical operation, tooth extraction, retinal photocoagulation or diabetic patients using steroid hormone.

Case 2 (Supplementary Fig. S2) is a patient with newly diagnosed T2DM. He had obesity and fatty liver. He succeeded weight control, recovery from fatty liver and glycaemic control after SIIT and maintenance medication. As shown in Supplementary Fig. S2, widely distributed blood glucose levels (8.3–14.4 mmol/L) converged to the target range of 5.0–7.2 mmol/L without hypoglycaemia. After discharge, fatty liver was ameliorated together with marked reduction of ALT level (Supplementary Fig. S2). Two causes of insulin resistance, obesity and fatty liver, were eliminated by SIIT and maintenance therapy.

Case 3 (Supplementary Fig. S3) was diagnosed as T2DM 16 years before, and had been treated with basal-bolus insulin therapy for 13 years. After SIIT, this patient discontinued insulin therapy and took maintenance therapy. High C-peptide and high HOMA-IR suggest that large amount of insulin secreted from beta-cells did not compensate strong insulin resistance. Massive (Mix-Insulin 162 units) and longer (FRD 16 days) SIIT served additional insulin to suppress blood glucose level, leading to relief of glucotoxicity and enhancement of insulin sensitivity. As seen in this case, 11 patients among 16 patients using insulin before SIIT succeeded to stop insulin and got glycaemic remission with subsequent duraglutide and NIDA. These data suggest that this method is also useful for the patients with inadequately controlled diabetes in spite of long-term insulin therapy.

In Case 4 (Supplementary Fig. S4), using continuously insulin therapy without OADs, after SIIT, each insulin becomes stable, adjusting to the target glucose levels. This patient was able to regulate insulin dose after discharge from the hospital. Supplementary Fig. S5 shows a typical common profile, and Supplementary Figs. S6 and S7 reveal profiles with higher dose of morning glulisine and no noon glulisine, suggesting that a premixed insulin with twice injection a day is suitable for these patients. Supplementary Figs. S8 and S9 unexpectedly present profiles with higher dose of three glulisine and no Gla-300. This is reproducible in a case and sometimes observed in thin old patients. We have to remember such cases because it seemed to be difficult to find these cases by using usual basal bolus injection.

Insulin resistance induces NAFLD and NASH as a complication of T2DM^48^. SIIT relieves insulin resistance by eliminating glucotoxicity. It was supported by the rapid decline of insulin dosage from 7 day to 9 day. In maintenance therapy after SIIT, GLP-1 receptor agonist dulaglutide, metformin, and SGLT2 inhibitor empagliflozin are agents for treatment of fatty liver^48–52^. Voglibose and mitiglinide might contribute to the relief of NASH by subsiding post prandial hyperglycaemia. We think that maintenance therapy with such medicines would be favorable in subjects with NAFLD or NASH.

In most SIIT studies, they did not use any anti-diabetic agents after SIIT^3–18^. Retnakaran *et al*. proposed sequential anti-diabetic agents as a maintenance therapy after induction therapy SIIT because remission is temporary without anti-diabetic agents^21^. Several kinds of anti-diabetic agents such as repeated SIIT, metformin, exenatide and glargine had been used as a maintenance therapy^24–26^. We treated the patients with maintenance therapy after M-SIIT for application to various diabetic patients, using empagiflozin, linagliptin (or dulaglutide), metformin and a mixed tablet of mitiglinide and voglibose.

Final insulin dose of 4 insulin injections was set to appropriate dose independently in each patient and in each injection by N-SIIT method. This method has a benefit to control insulin-requiring patients as a maintenance therapy after SIIT induction therapy. This SIIT enabled us to converge widely distributed blood glucose levels into target blood glucose range. From another viewpoint of “treat” insulin dosage, three bolus and one basal inulin are independent of each other. In this method, we can set optimal dosage of insulin based on each patient’s own pathophysiological condition and/or insulin requirement (Supplementary Figs. S4–S9). In other words, insulin requirement in each patient is appropriately obtained by this method.

We would like to emphasize here that this method is pretty simple and cheap without using any special devices such as insulin pump or continuous blood glucose monitoring system and thus available and useful in various situations from the clinical point of view. For example, we think that this method would be very useful when we introduce insulin therapy for the first time and adjust insulin dosage in subjects with T2DM. It is likely that required amount of insulin would be almost automatically and more easily determined with this method compared to conventional one.

In conclusion, we devised a new method for short-term intensive insulin therapy, which enabled us to eliminate glucotoxicity with very low risk of hypoglycaemia. This new SIIT exerts beneficial effects on beta-cell recovery by reducing glucotoxicity and promoting beta-cell rest. This method is based on each patient’s own pathophysiology and life style. We can confirm the relief of insulin resistance as well as glucotoxicity from the profile of insulin shot and glucose levels during N-SIIT. This method is simple and flexible to adapt to various diabetic conditions and useful in clinical practice for multiple purposes for diabetes treatment.

## Supplementary information

Supplementary information.
