# Supplementary material for: Practical application of short-term intensive insulin therapy based on the concept of “treat to target” to reduce hypoglycaemia in routine clinical site
Source: Sci Rep. 2020 Jan 31;10:1552. doi: 10.1038/s41598-020-58574-7 (PMC6994695; doi:10.1038/s41598-020-58574-7)
Supplement: Supplementary file 1 — Supplementary information. [file 41598_2020_58574_MOESM1_ESM.pdf]

Supplementary Dataset

Practical application of short-term intensive insulin therapy based on the concept of “treat to target” to reduce hypoglycaemia in routine clinical site.

Koji Nakashima, Nobuhiro Okamura, Hayato Sanefuji and Hideaki Kaneto

Corresponding author. Koji Nakashima MD, PhD.
Department of Internal Medicine,
Okamura Isshindow Hospital,
2-1-7 Saidaiji-Minami, Okayama, Okayama 704-8117. Japan.
Phone (81-86) 942-9901; Fax (81-86) 942-9929
E-mail address: naka-jii@med.kawasaki-m.ac.jp

Supplementary Fig. S1

a

Case 1

Date	9/17	9/18	9/19	9/20	9/21	9/22	9/23	9/24	9/25	9/26	9/27
Days	1	2	3	4	5	6	7	8	9	10	11
Bef. Breakfast	15.2	14.2	13.6	9.9	7.3	7.9	6.3	6.8	5.6	6.3	5.6
Bef. Lunch	18.5	16.8	16.3	13.3	5.9	8.2	5.7	4.1	4.7	6.3	4.7
Bef. Dinner	17.4	16.8	15.3	12.7	10.1	5.5	4.7	5.2	6.3	7.0	5.1
Bef. Bedtime	24.1	17.3	17.8	13.8	11.8	5.5	4.5	3.7	8.3	6.4	4.2
Cla-300-E	12	18	22	26	26	28	28	28	28	28	28
Gluli-M	0	6	10	10	12	12	12	12	12	10	10
Gluli-N	0	6	10	10	12	14	14	14	12	10	8
Gluli-E	6	8	10	12	12	14	16	14	12	12	12
	ampicillin						cefdinir				

Date	9/28	9/29	9/30	10/1	10/2	10/3	10/4	10/5	10/6	10/7	10/8
Days	12	13	14	15	16	17	18	19	20	21	22
Bef. Breakfast	5.6	5.8	5.7	5.5	5.3	5.2	5.4	5.8	5.7	5.6	5.7
Bef. Lunch	6.8	5.3	4.8	5.3	4.9	5.3	5.1	6.7	5.7	6.6	5.8
Bef. Dinner	6.4	5.6	4.9	5.8	5.8	4.7	6.5	7.3	6.4	6.7	7.4
Bef. Bedtime	4.8	5.0	3.9	6.4	5.8	5.6	5.8	9.4	7.8	7.4	9.0
Cla-300-E	28	28	28	26	26	26	20	18	18	18	18
Gluli-M	8	8	8	6	6	4	4	0	0	0	0
Gluli-N	8	8	8	6	4	4	4	0	0	0	0
Gluli-E	12	12	12	10	8	8	6	0	0	0	0
	cefdinir					metformin + linagliptin					

b

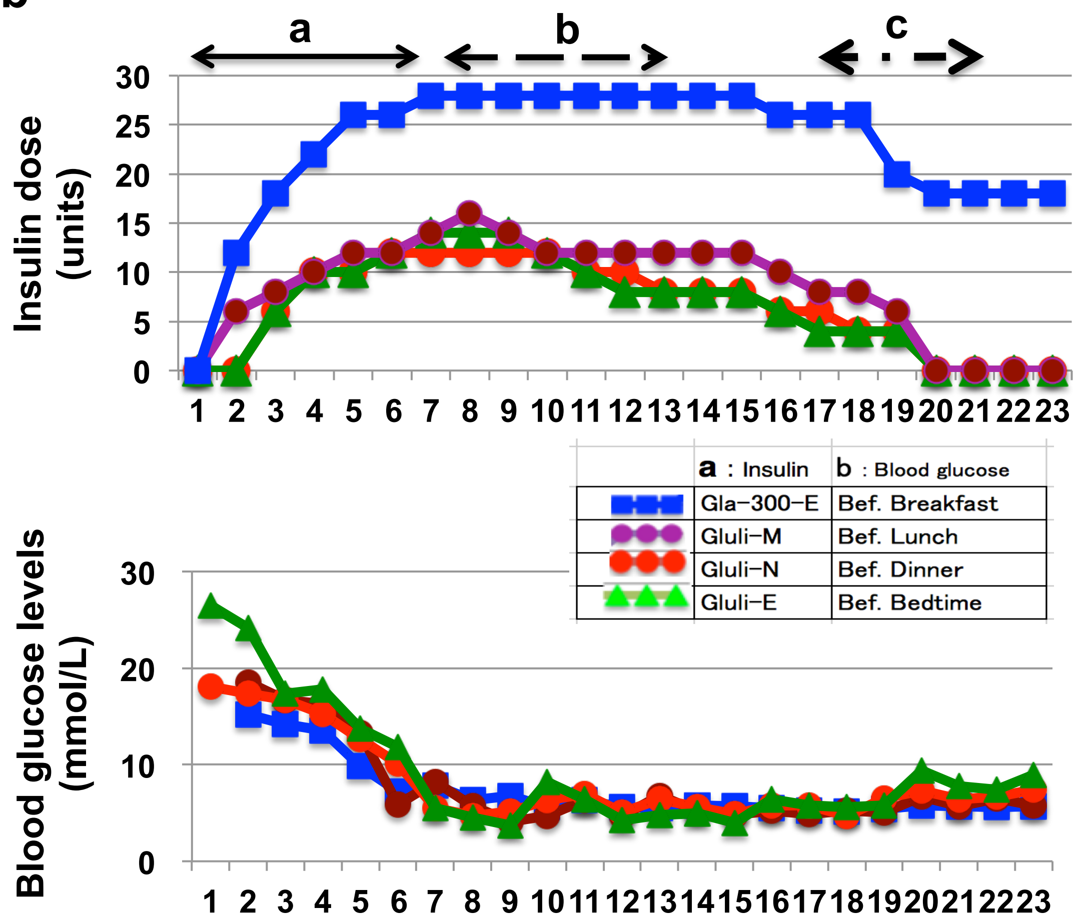

Supplementary Fig. S1

a. Insulin titration

Using our Case 1, we would like to explain the procedure of N-SIIT.. Blood glucose levels were recorded in the table after measurement to titrate “treat” insulin dose (a). In Table 1, dashed line was ordered by a doctor and solid line was performed by a nurse. Target level of blood glucose was 5.0-7.2 mmol/L. When blood glucose level before breakfast was higher than 7.2 mmol/L, two units were added to the yesterday’s Gla-300 to make today’s “treat” insulin. In the next day, when target glucose was still higher, 2 units were added again (Table 1, 9/18-9/22). It was repeated until when target blood glucose levels became less than 7.2 mmol/L. When blood glucose levels were within 5.0-7.2 mmol/L, the same dosage was injected (Table 1, 9/23-9/30). Like Gla-300 dose regulation, glulisine was injected before each meal until when each glucose level became within 5.0-7.2 mmol/L (Table 1, 9/21, 9/22, 9/26, 10/1). After most of blood glucose levels reached the target range, we stopped insulin and started non-insulin diabetic agents (NIDA)).

Case report

We received informed consent that we would like to publish the patient's history and their clinical and laboratory data in the worldwide journal, scientific reports from following cases.

b. Case 1

A 58-year-old house wife was admitted to our hospital because she developed cellulitis on the right wrist and forearm. She was diagnosed as diabetes mellitus 5 years before but she received no treatment. Data on admission were as follows: HbA1c 13.0 %, blood glucose 23.6 mmol/L, CP (C-peptide) 2.63 ng/ml (normal range: 0.61-2.09), CPI (C-peptide Index) 0.62 ng/mg (0.8-7.1), WBC 17,109 /μl, C-reactive protein (CRP) 16.8 mg/dl, body temperature 38.0°C. Staphylococcus aureus was grown from the sample of cellulitis. Ampicillin (2 g/day) was intravenously given and simultaneously N-SIIT was carried out. Insulin dose was adjusted as described in method. After treatment with ampicillin(a), oral cefdinir 300 mg/day (b) was given as a de-escalation of ampicillin. Prognosis of cellulitis and blood glucose control were satisfactory, and she discharged from the hospital on BOT (Basal-supported Oral Therapy) with Gla-300 18 u/day, linagliptin 5 mg/day, metformin 750 mg/day, mitiglinide 30 mg/day and voglibose 0.6 mg/day (c). After discharge, she titrated Gla-300 by measuring blood glucose levels by herself according to our protocol and reduced insulin dose step by step and discontinued Gla-300 one month later.

Supplementary Fig. 2

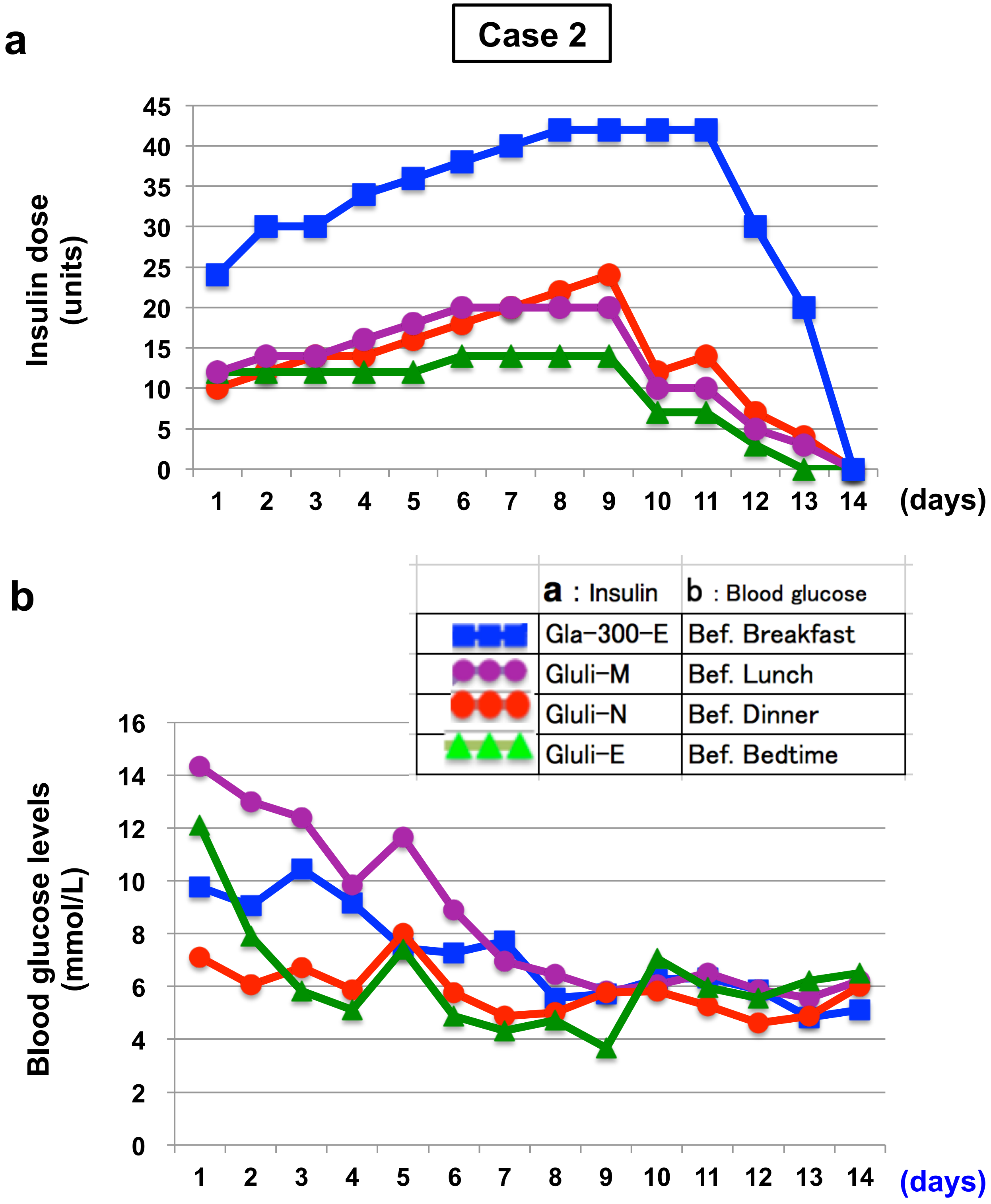

c

Case 2

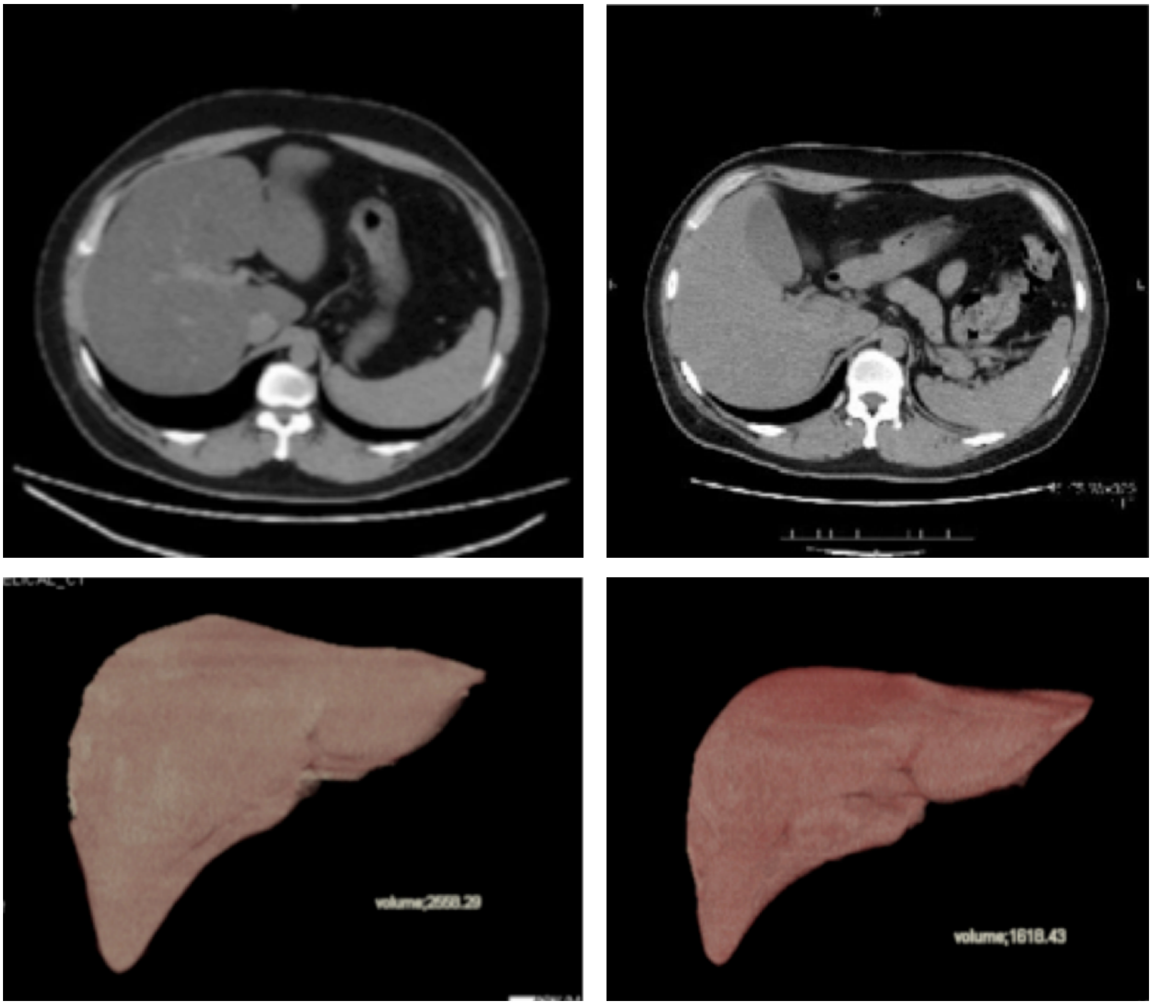

Abdominal CT

	Left picture : before SIIT	Right picture : after SIIT
Liver CT/spleen CT :	0.4	1.0
Liver volume :	2,558 ml	1,618 ml
subtraction		940 ml
ALT	231 U/L	10 U/L
HbA1c	9.2 %	5.4 %

Supplementary Fig. S2

Case 2

A 43-year-old male without history of diabetes (1 year before HbA1c was 5.9%) complained of dyspnea on exertion and was admitted to our hospital. ECG and chest X-ray were within normal range. Data on admission were as follows: blood glucose level was 12.7 mmol/L, HbA1c 9.2 %, AST 110 IU/l, and ALT 231 IU/l. Abdominal CT scan revealed fatty liver (liver CT/spleen CT ratio 0.4). The patient received N-SIIT and blood glucose decreased within the target range and insulin was replaced by dulaglutide 0.75 mg injection once/week and oral anti-diabetic medicine, empaglifrozin 10 mg/day, metformin 750 mg/day, mitiglinide 30 mg/day and voglibose 0.6 mg/day after discharge. He discharged from the hospital 14 days later and came to the out-patient clinic. Various markers were markedly improved as follows (2018/6/23 to 2018/11/9): body weight (101.6 kg to 72.8 kg), ALT (231 U/L to 10 U/L), blood glucose (13.3 mmol/L to 5.1 mmol/L), HbA1c (9.2% to 5.4%), L/S ratio (0.4 to 1.0), CT liver volume (2,558 ml to 1,618 ml).

Supplementary Fig. S3

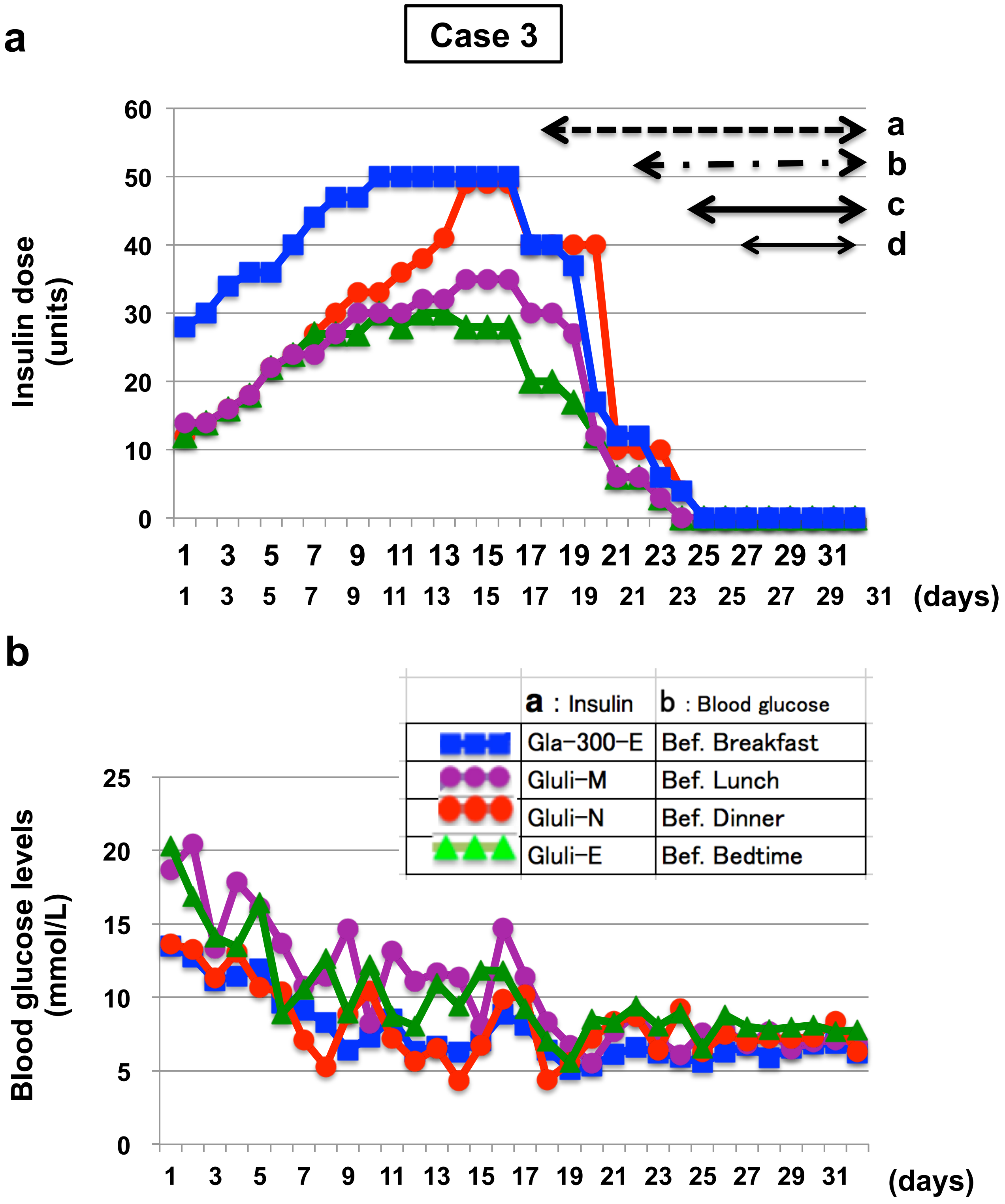

c

Case 3

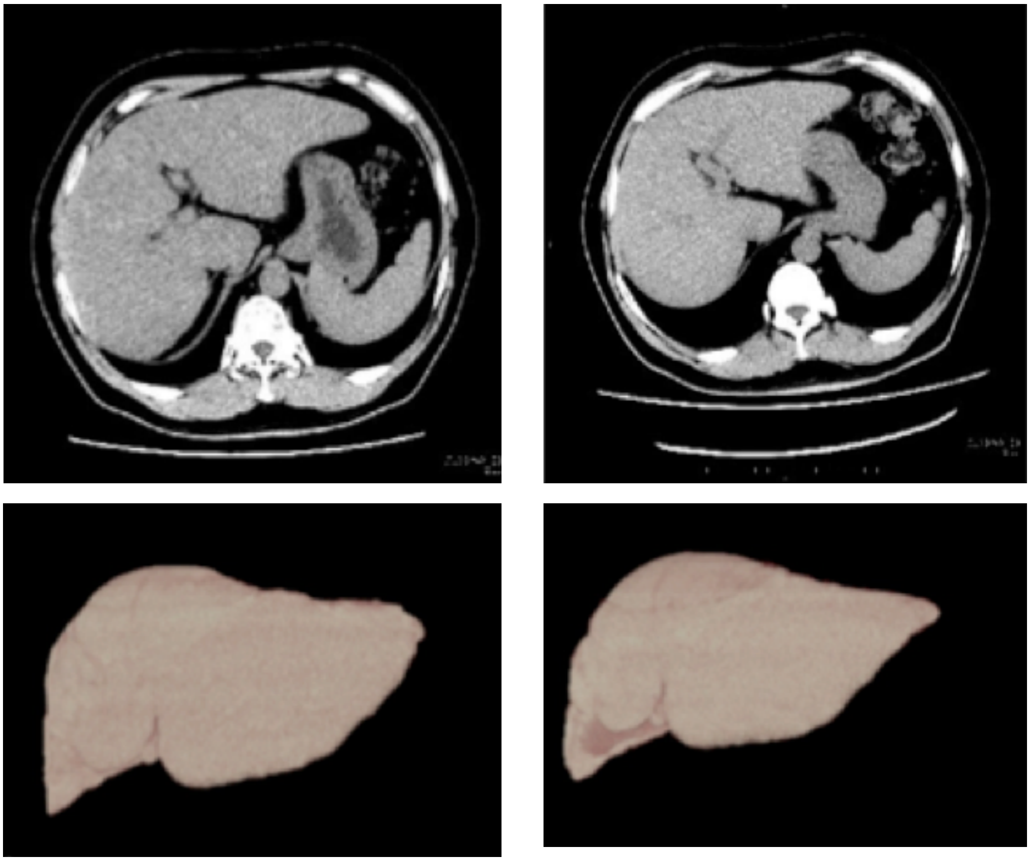

Abdominal CT

Left picture : before SIIT **Right picture : after SIIT**

	Left picture	Right picture
Liver CT/spleen CT :	0.8	1.0
Liver volume :	2,693 ml	1,886 ml
subtraction		807 ml
ALT	103 U/L	33 U/L
HbA1c	9.6 %	5.2 %

Supplementary Fig. S3

Case 3

A 51-year-old male with type 2 diabetes and fatty liver. Diabetes was diagnosed 16 years before, and basal-bolus insulin therapy was done for 13 years (insulin lispro 8 u, glargine 8 u), but HbA1c and blood glucose levels were high (HbA1c 9.6 %, FBS 13.4 mmol/L). This patient wanted to receive intensive insulin therapy and was admitted to our hospital. Insulin resistance was so strong that large amount of insulin (maximum total insulin 162 units/day) and 20 days of hospitalization were necessary. However, insulin was finally replaced by metformin 750 mg/day (a), mitiglinide 30mg/day (b), voglibose 0.6 mg/day (b), empagliflozin 10 mg/day (c) and dulaglutide 0.75 mg/week(d). Even after the replacement of high dose insulin to maintenance therapy, no rebound in glucose levels was observed, indicating the relief of insulin resistance. Various markers were markedly improved as follows: HOMA-IR: 4.5 (4/17), 2.3 (5/1), 1.3 (5/6); HbA1c levels: 9.6 (2018/4/3), 8.1 (5/1), 7.1 (5/18), 5.5 (7/13), 5.0 (2019/6/21); C-peptide: 3.54 (4/3), 1.6 (5/6).

Supplementary Fig. S4

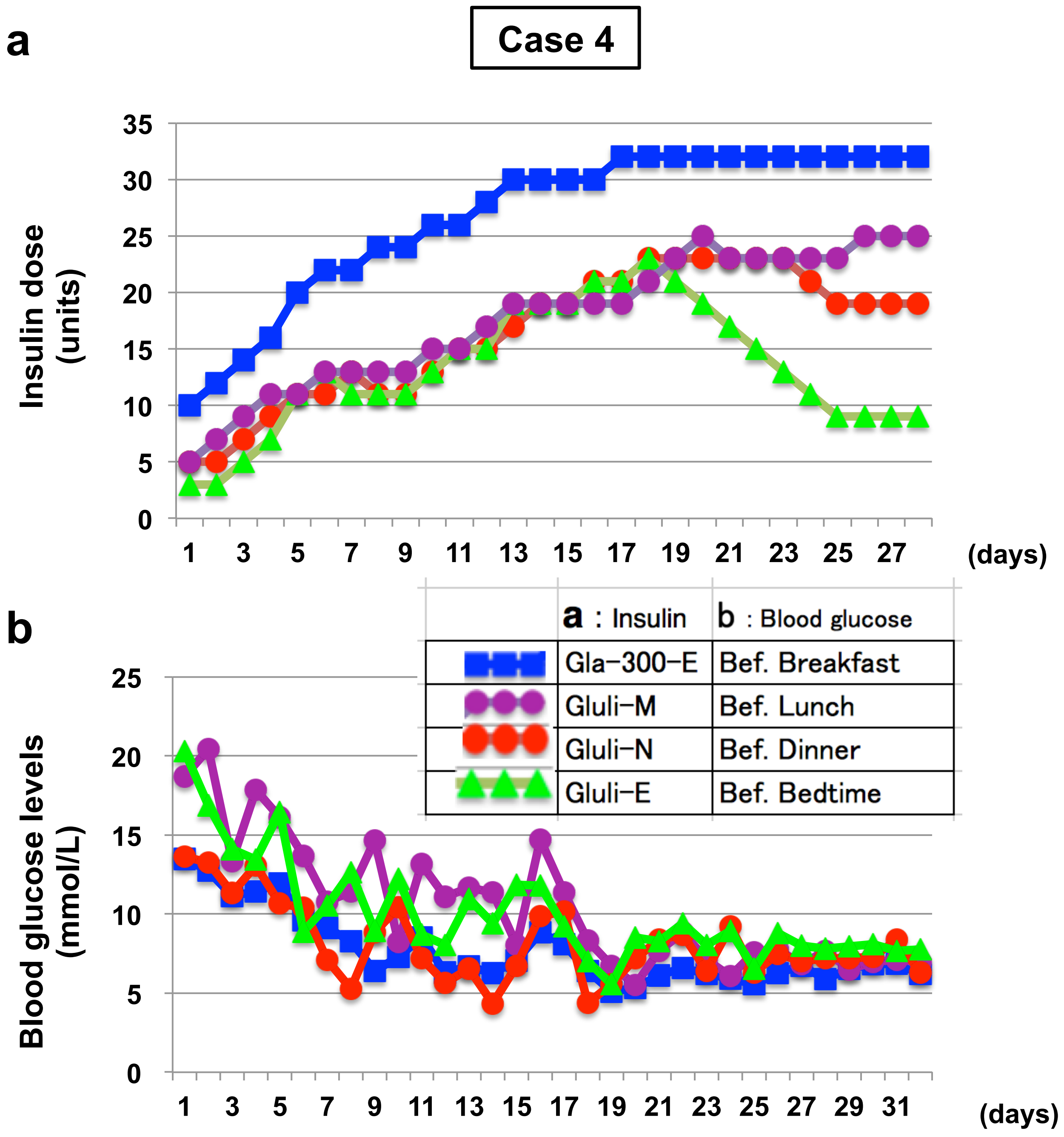

In poorly controlled patient with basal-bolus insulin therapy, SIIT was performed for reset of insulin therapy.

Upper graph : insulin dose (units)

Lower graph : blood glucose levels (mmol/L)

Supplementary Fig. S4

Case 4

A 62-year-old female with inadequately controlled T 2 DM was admitted to our hospital because she wanted to reset her glycaemic control. She had been treated with the basal-bolus insulin therapy for 15 years. Laboratory test showed HbA1c 8.9%, FBS 9.1 mmol/L, CP 0.14 mg/ml and CPI 0.17 μg/ml, indicating that she has exhausted beta cells and she needs insulin therapy for the maintenance of her life. We performed SIIT without any non-insulin diabetic agents (NIDA) as shown in Fig S4. Gla-300 and three Gluli dose was increased until day 19 when glucose levels converged into the target range. After day 19, dose of each insulin became stable, suggesting that this SIIT is a kind of procedure to determine appropriate insulin amount to keep target glucose levels. She received training of our protocol so that she was able to titrate her insulin dosage by herself after discharge. Before discharge, HbA1c was 7.1 %, FBS 5.2 mmol/L, CP 0.34 ng/ml and CPI 0.37 %, After discharge, she performed insulin regulation by herself. After 6 months, HbA1c was 6.4 %, FBG 5.9 mmol/L, CP 0.75 ng/ml and 0.71 ng/mg.

In the cases using insulin after N-SIIT (Supplementary Figs. S5-S9), we continued titration of insulin over two weeks, and insulin dose became stable in the stage within target glucose range. Supplementary Fig. S5 shows a typical common profile with high dose Gla-300 and low dose glulisine. Supplementary Figs. S6, S7 reveal profiles with higher dose morning glulisine and no noon glulisine. Supplementary Figs. S8, S9 present profiles with higher dose of three glulisine and no Gla-300. Final insulin dosage profiles were different from each other, suggesting that final insulin dose of 4 insulin injection was set to the appropriate dose independently in each patient and in each injection by N-SIIT method.

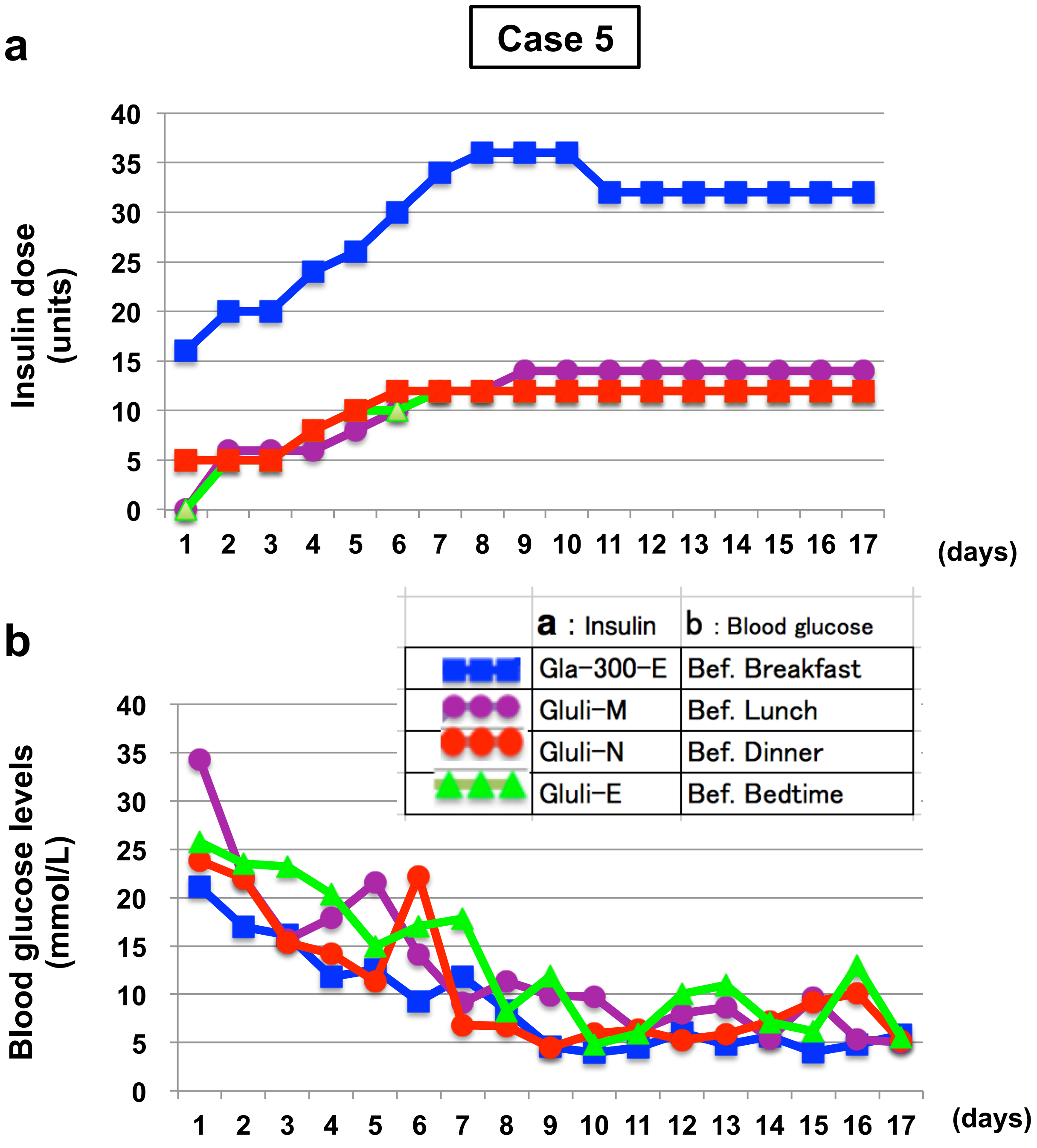

Profile of injected insulin and blood glucose level. 40-year-old male, T2DM, basal (0-0-32) – bolus(14-12-12) insulin therapy, HbA1c 14.8 %, FBS 38.7 mmol/L. N-SIIT was performed for reset of insulin therapy

Supplementary Fig. S6

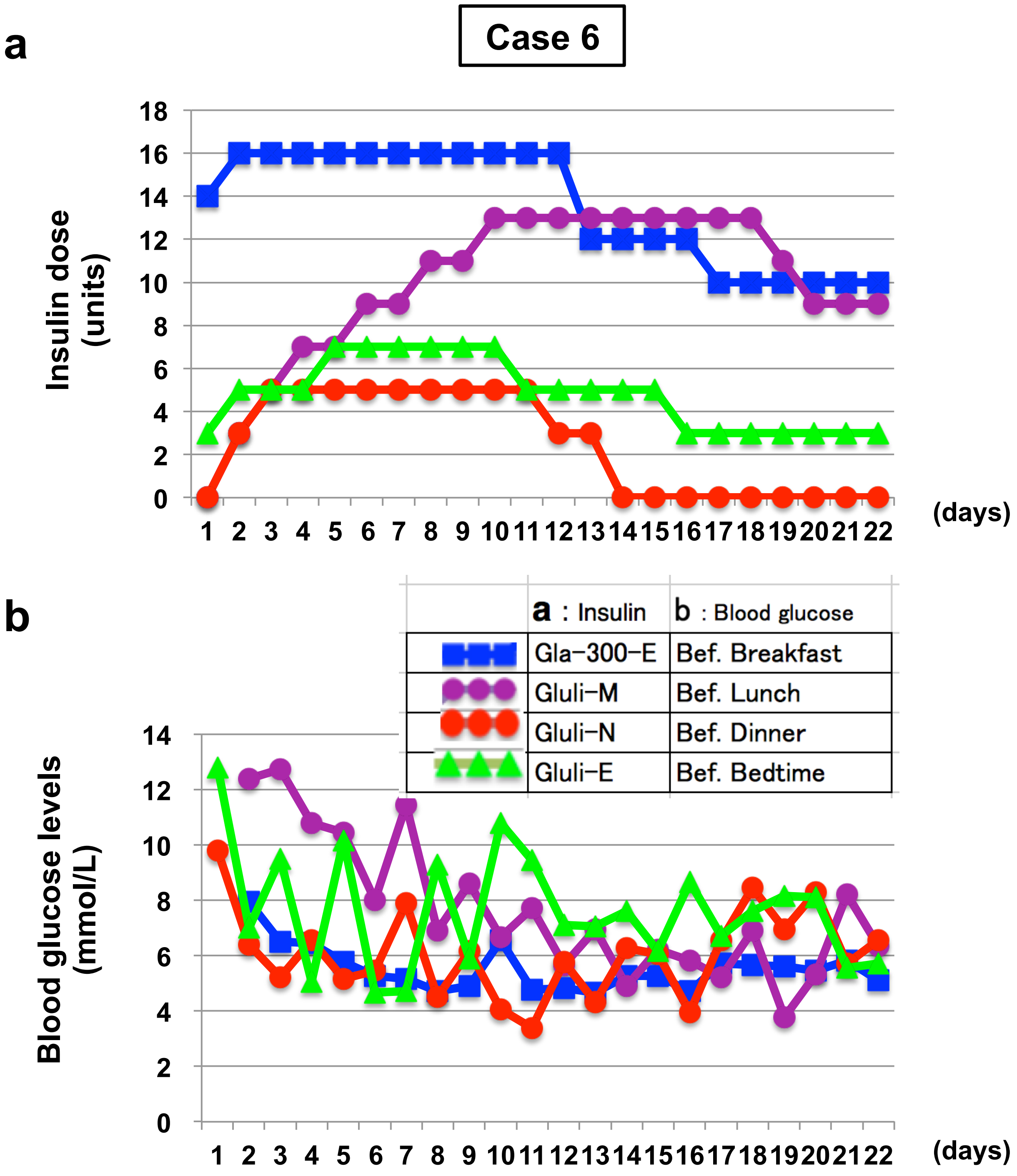

Profile of injected insulin and blood glucose level. 74-year-old male, T2DM basal (0-0-12) – bolus (3-3-3) insulin therapy, HbA1c 8.4 %, FBS 16.7 mmol/L, BMI 24.5 kg/m^2. N-SIIT was performed for reset of insulin therapy.

Supplementary Fig. S7

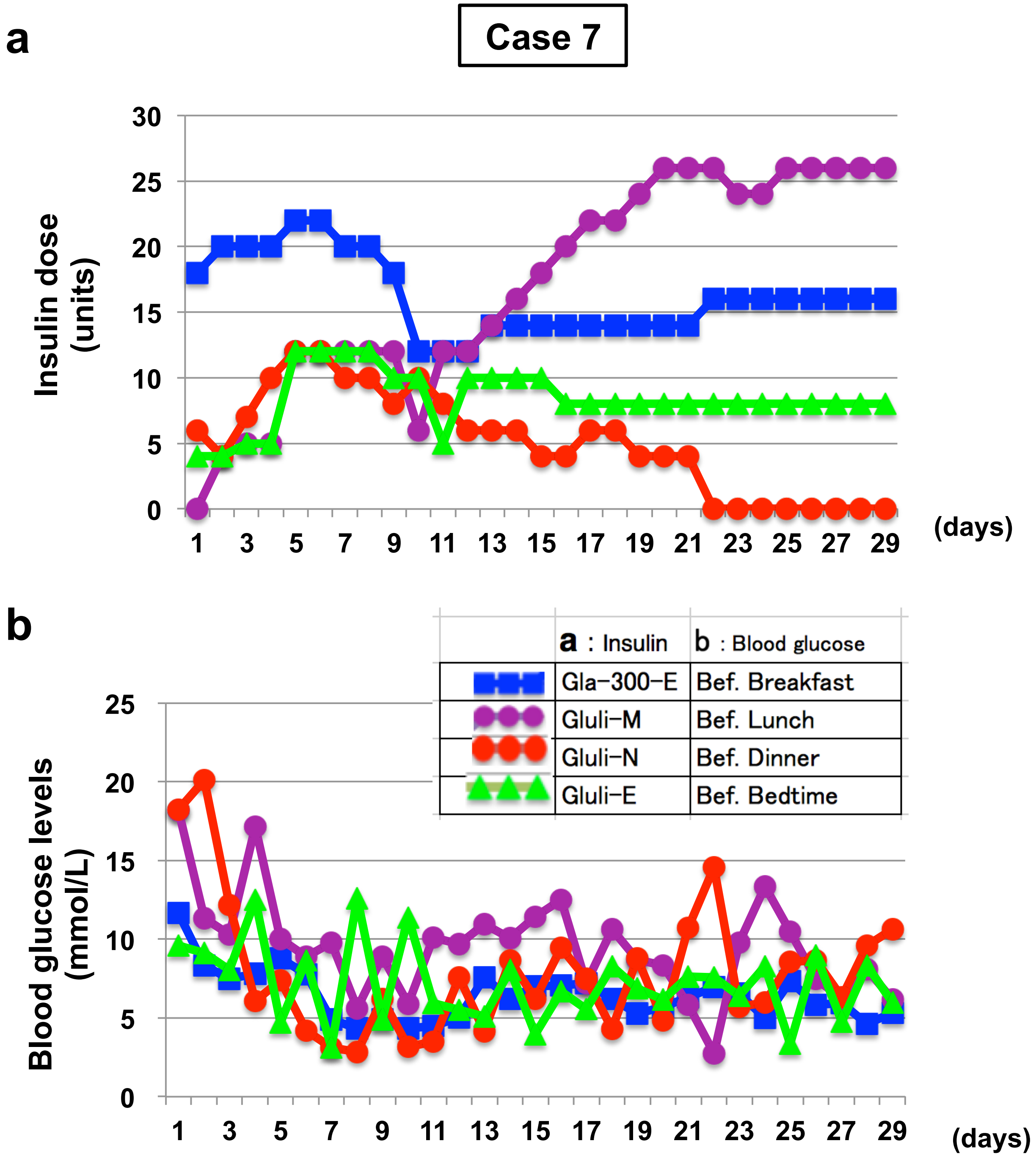

Profile of injected insulin and blood glucose level. 83-year-old female, T2DM, basal (0-0-12) – bolus (4-6-3) insulin therapy, HbA1c 10.4 %, FBS 18.1 mmol/L, BMI 21.2 kg/m^2. N-SIIT was performed for reset of insulin therapy.

Supplementary Fig. S8

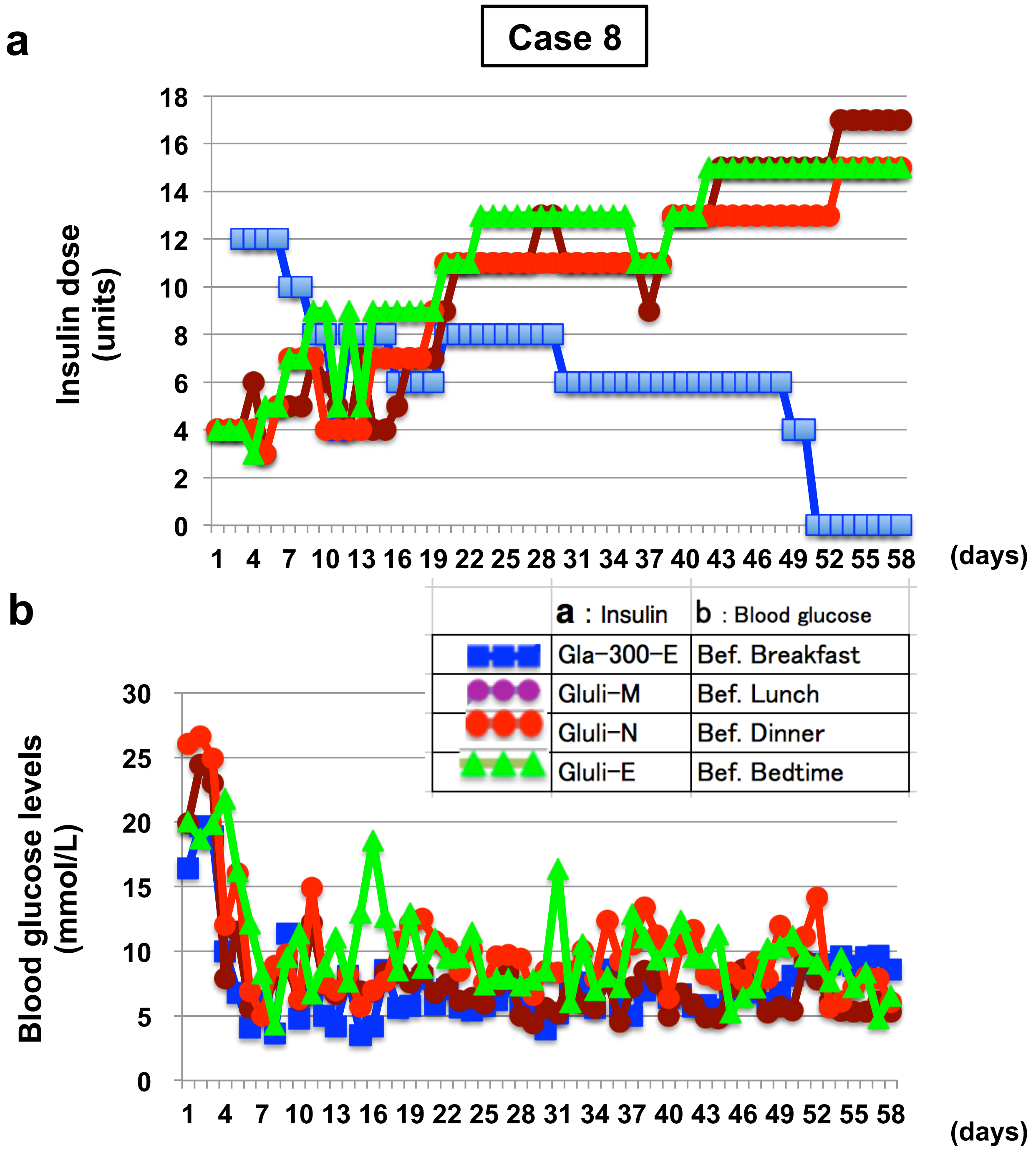

Profile of injected insulin and blood glucose level. 86-year-old female, T2DM, basal (0-0-12) – bolus (4-4-4) insulin therapy, HbA1c 8.0%, FBS 17.0 mmol/L, BMI 22 kg/m^2. N-SIIT was performed for reset of insulin therapy.

Supplementary Fig. S9

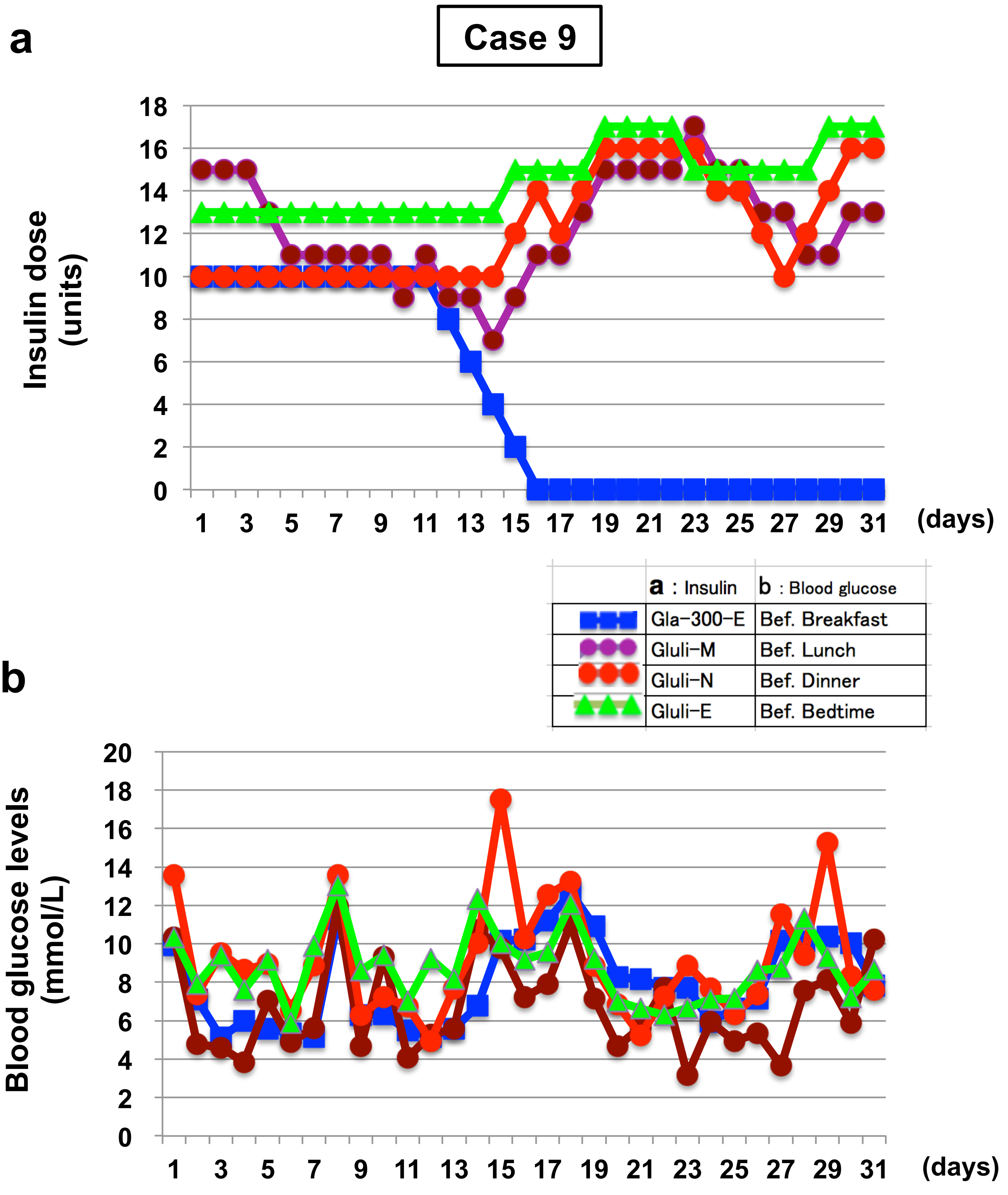

Profile of injected insulin and blood glucose level. 74-year-old male, T2DM, basal (0-0-12) – bolus (9-9-15) insulin therapy, HbA1c 8.0%, FBS 15.9 mmol/L, BMI 18.5 kg/m^2. H-SIIT was performed for reset of insulin therapy.

Supplementary Table T1

In C-SIIT, first insulin dose was determined by sliding scale.

average of pre-meal glucose mmol/L	Glargine units
<5.4	0
5.6-8.3	6
8.4-11.1	8
11.2-16.7	10
>16.7	12−16

(average of 2h PPG)-(average of premeal glucose) mmol/L	Lispro units
<2.8	4
2.9-8.3	6
8.4-11.1	8
>11.2	10

Glagine-100 was determined based on the average of three permeal blood glucose levels. Lispro was determined based on the [(average of 2h postprandial glucose) minus (average of premeal glucose)]

Three days later, Glargine was increased or reduced by 3 days average breakfast glucose. Lispro breakfast, Lispro lunch and Lispro dinner were determined by glucose lunch, glucose dinner and glucose bedtime respectively.

Six days later, the above was repeated.
